# Neurocognitive impairment and financial hardship in adult survivors of childhood cancer: a report from the Childhood Cancer Survivor Study

**DOI:** 10.1093/jnci/djaf383

**Published:** 2026-01-31

**Authors:** Daniel J Zheng, Shalini Bhatia, Sedigheh Mirzaei, Richard Aplenc, Gregory T Armstrong, Kira Bona, Tara M Brinkman, Pim Brouwers, Kim Edelstein, Kelly D Getz, I -Chan Huang, Anne C Kirchhoff, Sogol Mostoufi-Moab, Elyse R Park, Claire Snyder, K Robin Yabroff, Yutaka Yasui, Kevin Krull, Paul C Nathan

**Affiliations:** Division of Oncology, Children’s Hospital of Philadelphia, Philadelphia, PA, United States; St. Jude Children’s Research Hospital, Memphis, TN, United States; St. Jude Children’s Research Hospital, Memphis, TN, United States; Division of Oncology, Children’s Hospital of Philadelphia, Philadelphia, PA, United States; St. Jude Children’s Research Hospital, Memphis, TN, United States; Department of Pediatric Oncology, Division of Population Sciences, Dana-Farber/Boston Children’s Cancer and Blood Disorders Center, Boston, MA, United States; St. Jude Children’s Research Hospital, Memphis, TN, United States; National Institutes of Health, Washington, DC, United States; Princess Margaret Cancer Centre, Toronto, ON, Canada; Department of Biostatistics, Epidemiology and Informatics, University of Pennsylvania, Philadelphia, PA, United States; St. Jude Children’s Research Hospital, Memphis, TN, United States; Huntsman Cancer Institute, University of Utah, Salt Lake City, UT, United States; Division of Oncology, Children’s Hospital of Philadelphia, Philadelphia, PA, United States; Department of Psychiatry, Massachusetts General Hospital, Boston, MA, United States; Departments of Medicine, Oncology, and Health Policy & Management, Johns Hopkins University, Baltimore, MD, United States; Surveillance and Health Equity Science Department, American Cancer Society, Atlanta, GA, United States; St. Jude Children’s Research Hospital, Memphis, TN, United States; St. Jude Children’s Research Hospital, Memphis, TN, United States; Division of Haematology/Oncology, Department of Pediatrics, The Hospital for Sick Children, Toronto, ON, Canada

## Abstract

More than 60% of adult survivors of childhood cancer report medical financial hardship due to the cumulative costs of cancer-directed therapy and related chronic health conditions. Whether neurocognitive impairment as a late effect is associated with increased financial hardship is unknown. In a large national cohort of 3023 adult survivors of childhood cancer, we observed a dose-dependent relationship between neurocognitive impairment and financial hardship outcomes. Individuals with 4 impaired neurocognitive domains had over 3-fold increased odds of debt collection (odds ratio [OR] = 3.12, 95% confidence interval [CI] = 1.91 to 5.09) and bankruptcy (OR = 3.77, 95% CI = 2.01 to 7.07) compared to individuals with no impaired neurocognitive domains. After adjusting for education, employment, and household income, the independent association between neurocognitive impairment and financial hardship outcomes persisted suggesting that other mechanisms may be at play. Neurocognitive impairment is an important risk factor for medical financial hardship in this patient population and should be considered for targeted screening and intervention.

Medical financial hardship following cancer treatment is generally evaluated in 3 domains: material (trouble paying out-of-pocket expenses), psychological (distress over care costs), and behavioral (delaying/forgoing care because of cost).^1^ More than 60% of adult survivors of childhood cancer report hardship in at least one domain.^2^ A clearer understanding of risk factors is critical to mitigating the development and impact of financial hardship. Specific health conditions (eg, myocardial infarction, peripheral neuropathy, subsequent neoplasm, stroke, gastrointestinal disease) are associated with greater financial hardship among childhood cancer survivors.^3^ However, to date, the association between neurocognitive impairment and financial hardship has not been examined despite its high prevalence as a late effect with estimates as high as 40-50%.^4-6^ In this study, we investigated associations between neurocognitive impairment and financial hardship in a large cohort of adult survivors of childhood cancer and explored whether the association is moderated by education, employment, and income.

This was a retrospective cohort study leveraging data from the Childhood Cancer Survivor Study (CCSS). The CCSS has been extensively described in prior publications.^7^^,^^8^ Briefly, the CCSS is a large NCI-funded retrospective cohort of ≥5-year childhood cancer survivors diagnosed before 21 years of age between 1970 and 1999 at 31 participating institutions with longitudinal follow-up. Local institutional review boards at each of the participating institutions approved the study, and participants provided informed consent. For this analysis, we included US participants who completed a financial hardship questionnaire at age ≥26 years old and who had also completed a prior neurocognitive questionnaire (NCQ). The financial hardship questionnaire was offered to a randomly selected third (*n* = 5488) of the entire CCSS cohort between 2017 and 2019.^2^ The NCQ was offered to the entire CCSS cohort between 2014 and 2016.

The primary outcomes for this analysis were financial hardship outcomes for each of 3 previously mapped domains of behavioral hardship, material hardship, and psychological hardship, as well as 2 discrete items (sent to debt collection and filing for bankruptcy) that did not map onto any specific domain. Debt collection and bankruptcy in other studies have been categorized as part of an additional dimension of financial hardship termed “financial consequences.”^9^ Consistent with prior CCSS publication, we operationalized the financial hardship domain outcomes 2 ways: (1) binary scoring for any affirmative response to an individual item in each domain; and (2) standardized domain scores defined as the unweighted summation of affirmative responses in each domain divided by the standard deviation.^2^ For descriptive analyses, we described the binary scoring. For the primary adjusted analysis, we examined standardized mean differences (ie, differences in mean standardized domain scores).

The primary exposure of interest was neurocognitive impairment as measured by the validated patient-reported CCSS-NCQ and operationalized for this analysis as number of domains (0-4) impaired.^10^ The NCQ has items related to the specific domains of task efficiency, memory, organization, and emotional regulation. Consistent with prior publications, we defined impairment on a domain as a Z score >1.28, which corresponds to the worst tenth percentile of scores based on healthy control age-adjusted normative values.^10^^,^^11^

Covariates included age at financial hardship survey completion (continuous), sex (female vs. male), race/ethnicity (non-Hispanic White vs. other), anthracycline exposure (yes/no), alkylating agent exposure (yes/no), stem cell transplant (yes/no), non-cranial radiation therapy (yes/no), education (less than high school vs. less than college vs. college graduate/post-graduate), employment (employed full time/part time vs. unemployed), and household income (<$20 000 vs. $20 000-$59 999 vs. >$60 000). Education, employment, and household income were self-reported in the same survey and timepoint assessing financial hardship.

We described demographic and disease/treatment characteristics of the analytic cohort, as well as proportions reporting any affirmative response in each financial hardship domain, debt collection, and bankruptcy. In our primary analysis, for each financial hardship domain, we calculated standardized mean differences (SMDs) with the reference group being patients with no neurocognitive impairments using multivariable linear regression models. For debt collection and bankruptcy, we utilized multivariable log binomial models. Models were adjusted for a priori decided variables including age, sex, race/ethnicity, anthracycline exposure, alkylating agent exposure, stem cell transplant, and non-cranial radiation therapy.

We did not adjust for diagnosis and cranial radiation, given collinearity with the exposure of interest (neurocognitive impairment). We also did not adjust for education, employment, and household income in our primary model, as these factors were hypothesized to lie on the causal pathway and overadjustment would lead to an underestimate of the association. Instead, we subsequently evaluated the potential contributions of these factors to the relationship between neurocognitive impairment and financial hardship by secondarily adding them individually and altogether to the primary model.

The analytic cohort comprised 3023 adult (median age at financial hardship survey 40.5 [IQR 35.0-47.8] years) survivors of childhood cancer with the most common diagnoses being leukemia (31%), central nervous system tumor (16%), and Hodgkin lymphoma (13%). Forty-seven percent of patients had received anthracyclines, 51% had received alkylating agents, and 57% had received radiation therapy (27% had received cranial radiation and 2% had received total body irradiation). Most survivors (90%) were non-Hispanic White, had graduated college (60%), were employed (75%), and had private insurance (81%).

Overall, 31% of survivors had ≥1 domain of neurocognitive impairment with 14%, 8%, 6%, and 3% of survivors having 1-4 domains of neurocognitive impairment, respectively. Compared to survivors without neurocognitive impairment, survivors with 4 impaired neurocognitive domains were more likely to report ≥1 item in behavioral (62% vs. 25%), material (64% vs. 38%), and psychological (63% vs. 31%) domains of medical financial hardship. Similar patterns were observed for debt collection (54% vs. 25%) and bankruptcy (21% vs. 8%).

The SMDs for behavioral and material hardship increased with each unit increase in the number of neurocognitive domains impaired (Figure 1). Odds ratios for debt collection and bankruptcy showed the same dose-dependent pattern. Individuals with 4 impaired neurocognitive domains had over 3-fold increased odds of debt collection (odds ratio [OR] = 3.12, 95% confidence interval [CI] = 1.91 to 5.09) and bankruptcy (OR = 3.77, 95% CI = 2.01 to 7.07) compared to individuals with no impaired neurocognitive domains. After adjusting for education, employment, and household income, the independent association between neurocognitive impairment and financial hardship persisted across all hardship domains and both binary outcomes (Table 1).

**Figure 1.**
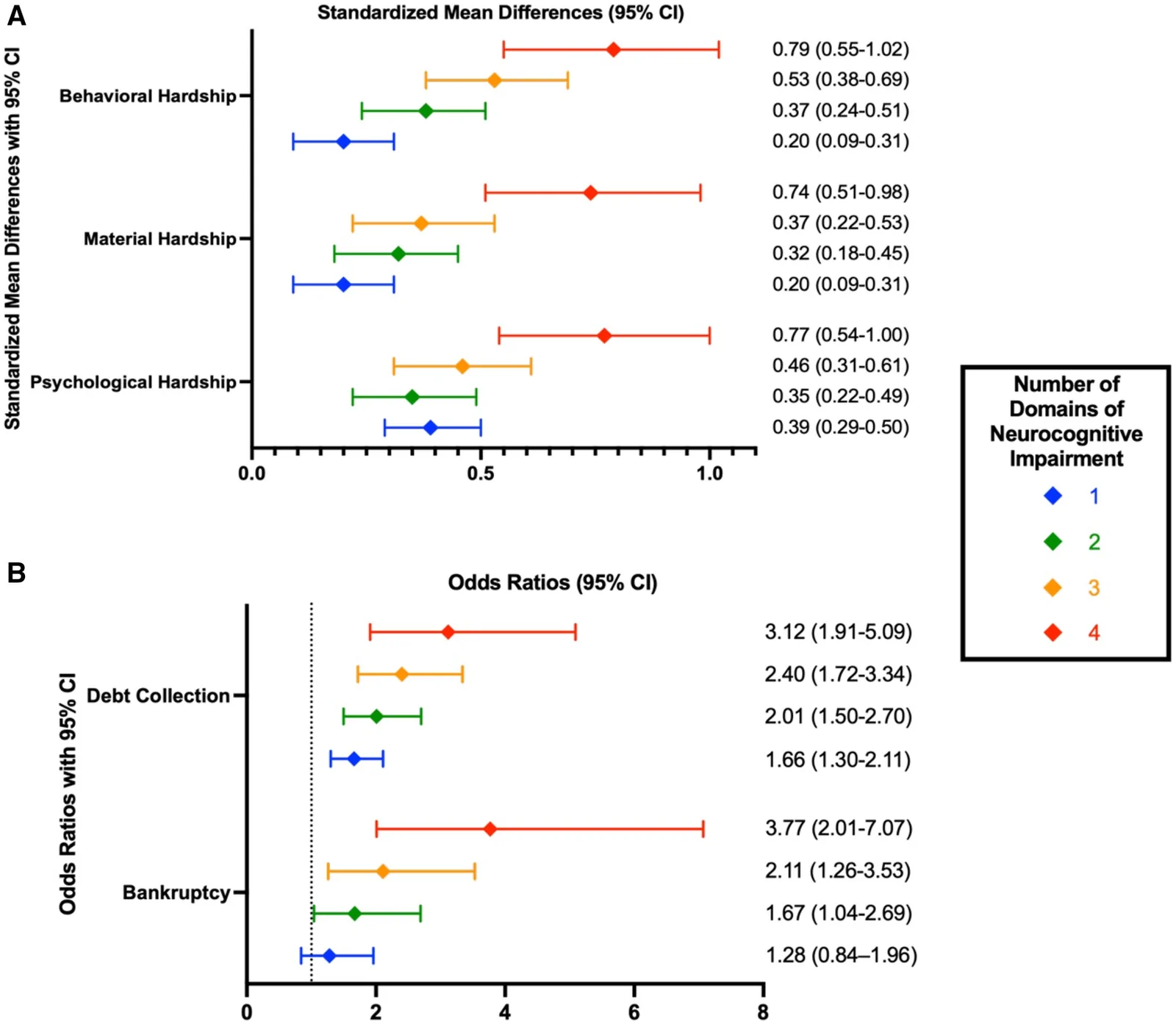
Standardized mean differences **(A)** and odds ratios **(B)** for financial hardship domains and outcomes by neurocognitive impairment compared to reference group of survivors with no neurocognitive domains impaired. Models adjusted for age at financial hardship survey, sex, race/ethnicity, and treatment exposures (ie, receipt of transplant, anthracycline, alkylating agent, non-cranial radiation).

**Table 1.** Multivariable adjusted associations of neurocognitive impairment and financial hardship outcomes.

	Primary model	Primary model + education	Primary model + employment	Primary model + income	Primary model + education, employment, and income
Behavioral Hardship, SMD (95% CI)					
0 neurocognitive domains impaired	Ref	Ref	Ref	Ref	Ref
1 neurocognitive domain impaired	0.20 (0.09 to 0.31)	0.13 (0.02 to 0.24)	0.18 (0.07 to 0.28)	0.18 (0.07 to 0.29)	0.12 (0.01 to 0.23)
2 neurocognitive domains impaired	0.38 (0.24 to 0.51)	0.33 (0.20 to 0.47)	0.34 (0.20 to 0.47)	0.25 (0.11 to 0.38)	0.23 (0.09 to 0.37)
3 neurocognitive domains impaired	0.53 (0.38 to 0.69)	0.48 (0.31 to 0.64)	0.48 (0.32 to 0.64)	0.43 (0.27 to 0.58)	0.41 (0.24 to 0.57)
4 neurocognitive domains impaired	0.79 (0.55 to 1.02)	0.60 (0.36 to 0.85)	0.72 (0.48 to 0.95)	0.63 (0.40 to 0.86)	0.48 (0.23 to 0.72)
Material Hardship, SMD (95% CI)					
0 neurocognitive domains impaired	Ref	Ref	Ref	Ref	Ref
1 neurocognitive domain impaired	0.20 (0.09 to 0.31)	0.21 (0.09 to 0.31)	0.20 (0.10 to 0.31)	0.21 (0.10 to 0.32)	0.23 (0.12 to 0.34)
2 neurocognitive domains impaired	0.32 (0.18 to 0.45)	0.32 (0.18 to 0.46)	0.32 (0.19 to 0.46)	0.24 (0.11 to 0.38)	0.27 (0.13 to 0.41)
3 neurocognitive domains impaired	0.37 (0.22 to 0.53)	0.35 (0.19 to 0.52)	0.38 (0.22 to 0.54)	0.33 (0.18 to 0.49)	0.36 (0.20 to 0.53)
4 neurocognitive domains impaired	0.74 (0.51 to 0.98)	0.63 (0.39 to 0.88)	0.75 (0.52 to 0.99)	0.70 (0.47 to 0.93)	0.63 (0.39 to 0.88)
Psychological Hardship, SMD (95% CI)					
0 neurocognitive domains impaired	Ref	Ref	Ref	Ref	Ref
1 neurocognitive domain impaired	0.39 (0.29 to 0.50)	0.34 (0.24 to 0.45)	0.38 (0.28 to 0.49)	0.38 (0.27 to 0.48)	0.37 (0.26 to 0.47)
2 neurocognitive domains impaired	0.35 (0.22 to 0.49)	0.27 (0.13 to 0.40)	0.34 (0.20 to 0.47)	0.17 (0.04 to 0.30)	0.19 (0.06 to 0.32)
3 neurocognitive domains impaired	0.46 (0.31 to 0.61)	0.41 (0.25 to 0.56)	0.43 (0.28 to 0.59)	0.34 (0.19 to 0.48)	0.41 (0.25 to 0.56)
4 neurocognitive domains impaired	0.77 (0.54 to 0.99)	0.61 (0.37 to 0.85)	0.74 (0.51 to 0.97)	0.61 (0.39 to 0.83)	0.57 (0.34 to 0.81)
Debt Collection, OR (95% CI)					
0 neurocognitive domains impaired	Ref	Ref	Ref	Ref	Ref
1 neurocognitive domain impaired	1.65 (1.29 to 2.11)	1.47 (1.14 to 1.89)	1.61 (1.26 to 2.06)	1.62 (1.26 to 2.08)	1.50 (1.15 to 1.94)
2 neurocognitive domains impaired	2.01 (1.50 to 2.70)	1.78 (1.31 to 2.43)	1.93 (1.43 to 2.60)	1.62 (1.18 to 2.19)	1.60 (1.16 to 2.20)
3 neurocognitive domains impaired	2.39 (1.71 to 3.32)	2.02 (1.41 to 2.89)	2.25 (1.60 to 3.16)	2.04 (1.44 to 2.88)	1.95 (1.35 to 2.83)
4 neurocognitive domains impaired	3.07 (1.88 to 5.01)	2.96 (1.75 to 5.03)	2.86 (1.74 to 4.70)	2.53 (1.52 to 4.22)	2.86 (1.65 to 4.96)
Bankruptcy, OR (95% CI)					
0 neurocognitive domains impaired	Ref	Ref	Ref	Ref	Ref
1 neurocognitive domain impaired	1.28 (0.84 to 1.96)	0.97 (0.61 to 1.54)	1.33 (0.87 to 2.03)	1.29 (0.83 to 1.98)	1.01 (0.63 to 1.64)
2 neurocognitive domains impaired	1.67 (1.04 to 2.69)	1.53 (0.94 to 2.48)	1.76 (1.08 to 2.85)	1.54 (0.94 to 2.53)	1.56 (0.94 to 2.60)
3 neurocognitive domains impaired	2.11 (1.26 to 3.53)	1.98 (1.17 to 3.35)	2.27 (1.34 to 3.85)	2.13 (1.26 to 3.62)	2.29 (1.33 to 3.95)
4 neurocognitive domains impaired	3.77 (2.01 to 7.07)	3.65 (1.90 to 6.98)	4.12 (2.15 to 7.87)	3.65 (1.90 to 7.00)	4.33 (2.19 to 8.57)

Primary model adjusted for age, sex, race/ethnicity, bone marrow transplant, anthracycline, alkylating agents, and non-cranial radiation therapy. Education, employment, and household income were ascertained at the same time as financial hardship questionnaire.

Abbreviations: CI = confidence interval; OR = odds ratio; SMD = standardized mean difference.

In this large national cohort of over 3000 adult survivors of childhood cancer, we observed a strong dose-dependent relationship between neurocognitive impairment and medical financial hardship. Strikingly, 54% of survivors with 4 impaired neurocognitive domains had been sent to debt collection and 21% had filed for bankruptcy. These findings underscore the potentially severe implications of neurocognitive impairment as a late effect of cancer therapy on long-term psychosocial outcomes.

We interestingly observed a persistent association of neurocognitive impairment with financial hardship independent of education, employment, and income, suggesting other mechanisms may be at play. Neurocognitive impairment may exacerbate survivors’ difficulties navigating ongoing health costs in a complex healthcare system. There is current work exploring financial and health insurance navigation as potential interventions to mitigate financial burden in childhood cancer survivors.^12-15^ Another contributing mechanism may be the importance of higher cognitive function for appropriately managing one’s finances. Work in older adults with dementia and other cognitive impairments has demonstrated that patients with mild cognitive impairment have significant decline in financial skills over time, self-report greater difficulty in managing their finances, and needed more time and were significantly more likely to make an error when given a multistep financial task.^16-19^ While this population is not entirely analogous to adult survivors of childhood cancer (who may not be experiencing decline of financial management skills but rather never develop these skills in the first place), these findings point toward a need for proactive financial planning for this patient population. Given the unique lifelong care continuum for childhood cancer survivors, we ought to consider dual but complementary approaches of interventions for caregivers during childhood cancer therapy and for survivors themselves as they reach adulthood. In addition to financial hardship interventions, we also need to focus on minimizing the risk of long-term neurocognitive impairment from childhood cancer treatment through minimizing cumulative anesthesia exposure, de-escalation trials, and the development of neuro-protective interventions.^20-23^

These findings must be interpreted in the context of its limitations including that the CCSS cohort may not be entirely generalizable to a wider population particularly given limited diversity with regards to race/ethnicity. The data are also limited as a cross-sectional evaluation of financial hardship, precluding formal causal mediation analyses. Longitudinal assessments should be considered in the future. Finally, the employment and income data are self-reported and limited in detail which may restrict our ability to identify more nuanced relationships between these covariates and our outcomes of interest.

In summary, neurocognitive impairment is a strong independent risk factor for medical financial hardship in adult survivors of childhood cancer. This late effect warrants consideration of future targeted screening and potential intervention to improve long-term psychosocial outcomes for these patients.

## Data Availability

The Childhood Cancer Survivor Study is a US National Cancer Institute funded resource (U24 CA55727) to promote and facilitate research among long-term survivors of cancer diagnosed during childhood and adolescence. CCSS data are publicly available on dbGaP at https://www.ncbi.nlm.nih.gov/gap/ through its accession number phs001327.v2.p1. and on the St. Jude Survivorship Portal within the St. Jude Cloud at https://survivorship.stjude.cloud/. Utilization of the CCSS data that leverages the expertise of CCSS Statistical and Survivorship research and resources will be considered on a case-by case basis. For this utilization, a research Application Of Intent followed by an Analysis Concept Proposal must be submitted for evaluation by the CCSS Publications Committee. Users interested in utilizing this resource are encouraged to visit http://ccss.stjude.org. Full analytical data sets associated with CCSS publications since January of 2023 are available on the St. Jude Survivorship Portal at https://viz.stjude.cloud/community/cancer-survivorship-community∼4/publications.
